# Steroid-resistant nephrotic syndrome as the initial presentation of nail-patella syndrome: a case of a *de novo* LMX1B mutation

**DOI:** 10.1186/s12882-017-0516-7

**Published:** 2017-03-23

**Authors:** Tomohiro Nakata, Ryo Ishida, Yuu Mihara, Atsuko Fujii, Yoshimoto Inoue, Tetsuro Kusaba, Tsuyoshi Isojima, Yutaka Harita, Chiaki Kanda, Sachiko Kitanaka, Keiichi Tamagaki

**Affiliations:** 1 Division of Nephrology, Department of Medicine, Kyoto Prefectural University of Medicine, 465 Kajii-cho, Kamigyo-ku, Kyoto, 602-8566 Japan; 2 Division of Nephrology, Kyoto Min-iren Chuo Hospital, 16-1 Nishinokyo Kasuga-cho, Nakagyo-ku, Kyoto, 604-8453 Japan; 3 Department of Pediatrics, Graduate School of Medicine, The University of Tokyo, 7-3-1 Hongo, Bunkyo-ku, Tokyo, 113-8655 Japan

**Keywords:** Nail-patella syndrome, LMX1B, Steroid-resistant nephrotic syndrome

## Abstract

**Background:**

Nail-patella syndrome (NPS) is an autosomal dominant disorder caused by mutations in the LMX1B gene and is characterized by nail dysplasia, skeletal abnormalities, and nephropathy. We herein report a case of steroid-resistant nephrotic syndrome (SRNS) prior to overt orthopedic symptoms in a patient with NPS.

**Case presentation:**

A 24-year-old woman presented to our hospital with knee pain. She had poorly developed nails, hypoplastic patellas, dislocation of the elbows, and iliac horns in the pelvis. At the age of 7, she developed nephrotic syndrome and was diagnosed with primary focal segmental glomerulosclerosis by renal biopsy. She received long-term corticosteroid therapy with no obvious response. Her clinical course and orthopedic manifestations indicated NPS, and a genetic analysis showed a de novo mutation in the LMX1B gene (c.819 + 1G > A). Nephropathy in this case was considered to be associated with NPS. Therefore, we discontinued corticosteroids without the exacerbation of nephrotic syndrome.

**Conclusions:**

Patients with NPS may develop nephrotic syndrome prior to overt orthopedic symptoms and only show non-specific findings in renal biopsy at an early stage of NPS nephropathy. Hereditary nephrotic syndrome, often presenting as childhood-onset SRNS, may also be difficult to diagnose in patients with the following conditions: renal symptoms prior to overt extrarenal symptoms, de novo mutations, and non-specific findings in renal biopsy. Therefore, in the management of SRNS in children, we need to reconsider the possibility of hereditary diseases such as NPS even without a family history.

## Background

Nail-patella syndrome (NPS; OMIM 161200) is a pleiotropic autosomal dominant disorder characterized by nail dysplasia, patellar aplasia or hypoplasia, elbow dysplasia, and iliac horns in the pelvis [1–3]. The causative gene of NPS has been identified as the LIM homeobox transcription factor 1 beta (LMX1B; OMIM 602575) gene, which is located on the long arm of chromosome 9 [4]. The incidence of NPS was previously reported to be approximately 1:50,000, with de novo mutations accounting for 12.5% of NPS cases [4, 5].

Nail anomalies have been detected in 95.1% of NPS patients, patellar aplasia or hypoplasia in 92.7%, iliac horns in 70%–80%, elbow dysplasia in 92.5%, ocular hypertension in 7%, and glaucoma in 10% [3]. The incidence of renal involvement was found to be 37.5% in NPS patients, and the mean age when renal involvement was detected was 21.7 years [2]. Nephrotic syndrome is exceptional; most patients present with proteinuria and hematuria [2, 6]. Renal failure occurs in between 5 and 14% of NPS patients [2].

We herein report a case of childhood-onset steroid-resistant nephrotic syndrome (SRNS) that was diagnosed with NPS based on delayed orthopedic symptoms and a genetic analysis following long-term corticosteroid therapy. We also discuss the need to reconsider hereditary diseases in the management of children with SRNS even without a family history.

## Case presentation

**Fig. 1:**
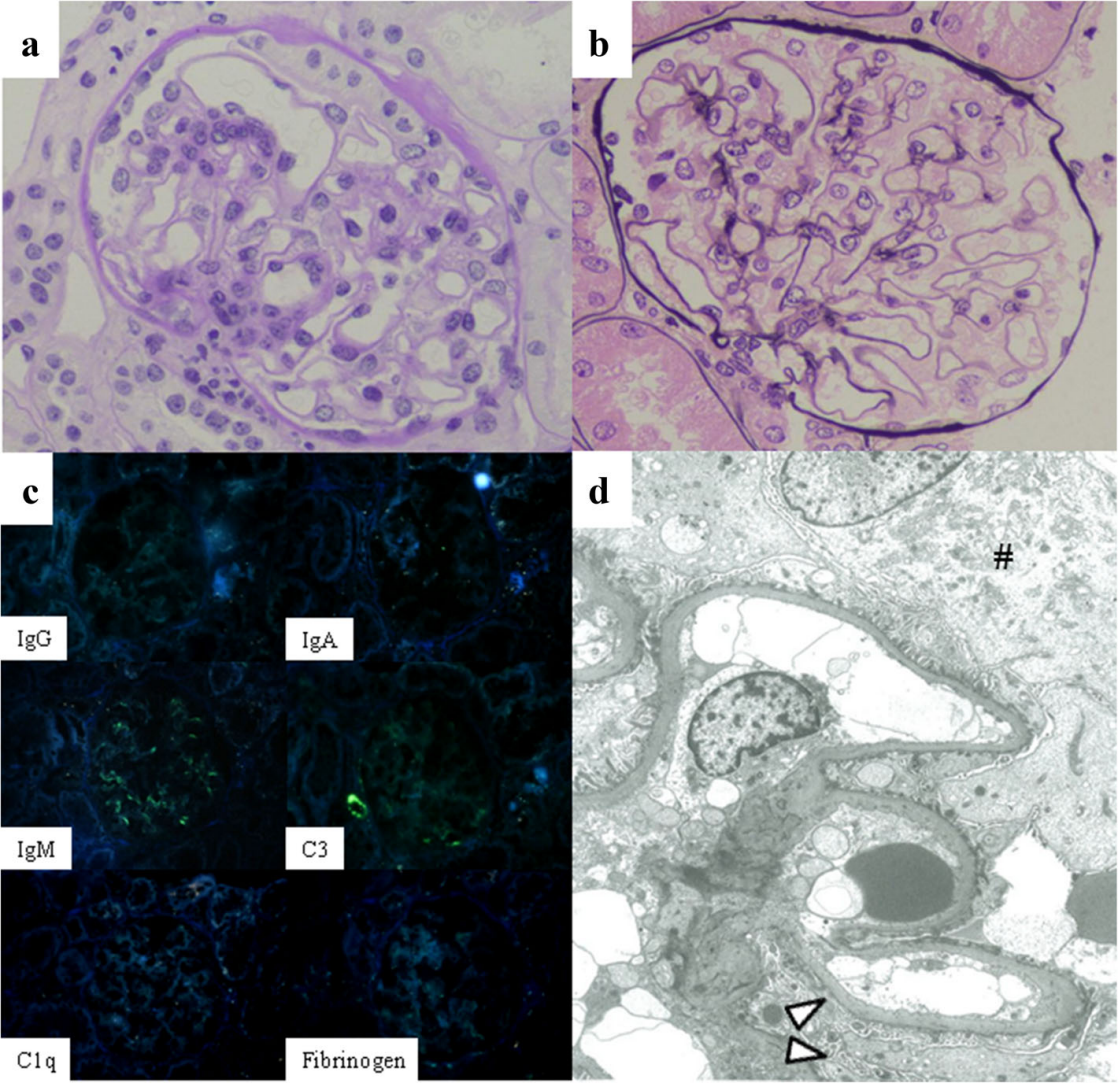
Renal biopsy at the age of 7. Light microscopy only showed focal glomerular lesions (**a**) Periodic-Acid Schiff stain x400, (**b**) Periodic acid-methenamine-silver stain x400). Immunofluorescence showed slightly positive staining for IgM and C3 within glomerular segmental lesions (**c**). Electron microscopy revealed mild irregular thickening of the GBM and swollen podocytes (# in **d**) with partial foot process effacement (arrowhead in **d**). Neither electron-lucent areas within the GBM nor electron-dense deposits were detected (**d**)

A 24-year-old woman presented to our hospital with pain in her knee. At the age of seven, she developed nephrotic syndrome and was diagnosed with primary focal segmental glomerulosclerosis (FSGS) by renal biopsy (Fig. 1). Nephrotic syndrome gradually worsened without symptoms such as edema. She was administered corticosteroids from the age of 18 years, but did not respond to this therapy. Cyclosporine was then administered, but was discontinued within one week due to the adverse effect of fatigue. Corticosteroids were tapered with proteinuria of 4 to 6 g per 24 hours.

**Fig. 2:**
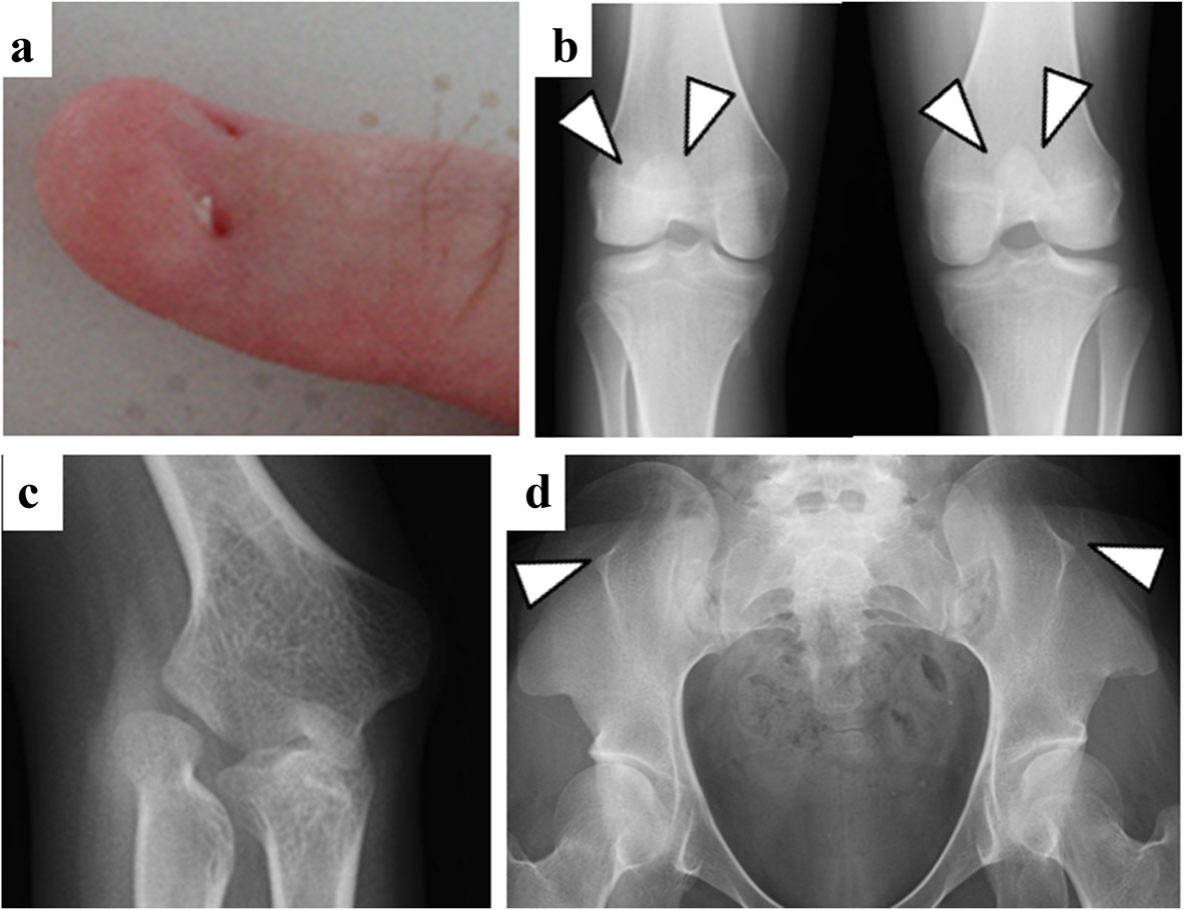
Nail abnormalities and orthopedic manifestations at the age of 24. She presented with poorly developed nails (**a**). An x-ray examination revealed hypoplastic patellas (**b**), dislocation of the elbows (**c**), and iliac horns in the pelvis (**d**)

**Fig. 3:**
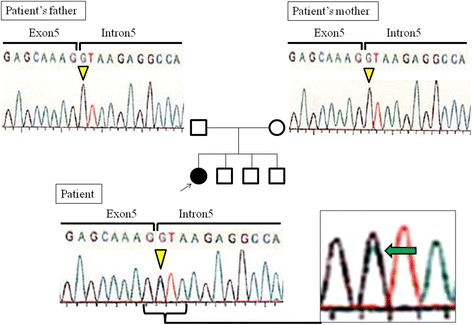
Genetic analysis. The genetic analysis showed a heterozygous mutation in the first base of the fifth intron (c.819 + 1G > A) of the LMX1B gene located on the long arm of chromosome 9. The same mutation was not detected in her parents

When she presented to our hospital, she had poorly developed nails and small corneas. Intraocular pressure was normal. She gradually developed pain in her knee over 12 months. X-ray and computed tomography were performed to systemically evaluate the orthopedic morphology, and revealed hypoplastic patellas, dislocation of the elbows, and iliac horns in the pelvis (Fig. 2). Laboratory data showed hypoalbuminemia and the excretion of a large amount of protein in the urine, indicating nephrotic syndrome with renal insufficiency. Other serological results revealed that the secondary causes of nephrotic syndrome such as autoimmune disease, hematological diseases, or infection were negative (Table 1). Her clinical course of SRNS and orthopedic manifestations indicated NPS; therefore, we performed a genetic analysis, which showed a heterozygous mutation in the first base of the fifth intron of the LMX1B gene (c.819 + 1G > A) (Fig. 3). Since this mutation was not detected in her parents, our case was considered to have a de novo mutation.

**Table 1:** Laboratory data on admission

Peripheral blood		Serological tests	
WBC	12100/μl	Anti-nuclear antibodies	Negative
RBC	515 × 10^4^/μl	Rheumatoid factor	Negative
Hb	14.8 g/dl	IgG	323 mg/dl
Ht	42.9%	IgA	179 mg/dl
Plt	43.3 × 10^4^/μl	IgM	259 mg/dl
Blood chemistry		C3	80.5 mg/dl
TP	4.8 g/dl	C4	14.6 mg/dl
Alb	2.8 g/dl	CH50	35.0 U/ml
AST	11 IU/ml	C1q	Negative
ALT	14 IU/ml	HBs antigen	Negative
LDH	240 IU/ml	Anti-HCV antibody	Negative
BUN	12.1 mg/dl	Urinalysis	
Cr	1.01 mg/dl	Gravity	1.013
eGFR	45 ml/min/1.73 m^2^	pH	6.5
Na	140 mEq/l	Protein	4+
K	3.6 mEq/l	Occult blood	-
Cl	105 mEq/l	Sediments	
LDL	123 mg/dl	RBC	<1/HPF
TG	157 mg/dl		
HbA1c	5.2%	Urinary chemistry	
CRP	0.01 mg/dl	Protein	7.67 g/gCr

In a review of renal biopsy at the age of 7, light microscopy revealed focal glomerular lesions only (Fig. 1a-b). Immunofluorescence showed slightly positive staining for IgM and C3 within glomerular segmental lesions (Fig. 1c). Electron microscopy displayed mild irregular thickening of the glomerular basement membrane (GBM) and swollen podocytes with partial foot process effacement, whereas distinctive lesions such as electron-lucent areas within the GBM were not detected (Fig. 1d). Although we were unable to perform renal biopsy again because of renal atrophy (parenchymal thinning, an indistinct corticomedullary junction, and longitudinal length of 9 cm by ultrasonography), nephropathy in this case was considered to be associated with NPS. Therefore, we discontinued corticosteroids gradually without the exacerbation of nephrotic syndrome.

## Discussion

NPS is an autosomal dominant disorder characterized by orthopedic manifestations. In our case, NPS was difficult to diagnose early for the following reasons: 1) nephrotic syndrome developed prior to overt orthopedic symptoms; 2) she had no family history; and 3) renal pathology revealed only non-specific findings in the GBM at an early stage of NPS nephropathy. Based on the presence of orthopedic symptoms, we performed a genetic analysis, detected a de novo mutation in LMX1B without this mutation in her parents, and discontinued unnecessary steroid treatments.

LMX1B is expressed in the dorsal mesenchyme of developing vertebrate limbs and plays a critical role in the patterning of the dorsal-ventral axis [7]. Once the dorsal-ventral axis is established, LMX1B activates the expression of a number of dorsalizing genes, suppresses the expression of ventralizing genes, and modulates the development of dorsal distal limb structures such as nails and patellas [4]. In addition to limb development, the LMX1B gene is associated with the development of multiple organs such as the brain, spinal cord, eyes, ears, and kidneys [8, 9]. In our case, this gene mutation was implicated in the impairments observed in the limbs, nails, and eyes as extrarenal symptoms.

The expression of LMX1B in the kidney was initially detected in the region of the S-shaped body, which gives rise to the glomerulus, and subsequently in the visceral epithelium of the glomerulus in experiments on mice [4]. LMX1B binds to an enhancer sequence in intron 1 of the collagen IV α4 chain and regulates the coordinated expression of collagen IV α3 and α4 chains, which are required for normal GBM morphogenesis [10]. A previous study on heterozygous LMX1B knockout mice demonstrated that LMX1B affects increases in glomerular volume as a compensatory response to the loss of nephrons [11]. Other studies on podocyte-specific LMX1B conditional knockout mice demonstrated that LMX1B is required for the initial differentiation of podocytes and development of foot processes [12, 13]. In addition, LMX1B is considered to bind to sites in the putative regulatory regions of the CD2AP and NPHS2 genes and regulate their expression, which is associated with the formation of the slit diaphragm [13].

More than 140 mutations have been detected in LMX1B, and the mutation in our case was previously reported to result in the loss of function of LMX1B due to a dysfunction in exon 6 [14]. Although comparisons of phenotypes with genotypes have not revealed any relationship between disease severity and the type or location of mutations [14], specific missense mutations in LMX1B have recently been identified in cases of nephropathy without extrarenal manifestations [15, 16]. In the present case, nephrotic syndrome developed at the age of 7, followed by overt orthopedic symptoms after the growth period. Therefore, our case is clearly distinguishable from previously reported cases of isolated nephropathy caused by LMX1B mutations.

The renal pathological findings of NPS nephropathy vary from minor glomerular abnormalities to FSGS under light microscopy [17], and irregular increases in the thickness of the GBM are observed with the progression of the disease. Immunofluorescence is typically negative or IgM and C3 are slightly positive within glomerular segmental lesions. In addition, electron microscopy shows irregular thickening of the GBM with electron-lucent areas, which is known as the moth-eaten appearance. The deposition of collagen III has been detected in GBM lesions [17]. However, the extent and distribution of GBM lesions vary individually without any relationship with the age of patients or severity of renal symptoms [17]. Thus, non-specific pathological findings may be observed at an early stage of NPS nephropathy. In our case, only focal segmental glomerular lesions and mild irregular thickening of the GBM without electron-lucent areas were detected. Consequently, she was not diagnosed with NPS at the age of 7, and received immunosuppressive therapy. Renal involvement in NPS is caused by a LMX1B gene mutation, which dysregulates the production of collagen, and corticosteroids have no effect on this gene mutation. Thus, her clinical course with no response to corticosteroids supported the diagnosis of NPS. There are currently no specific treatments for renal involvement in NPS, except for angiotensin-converting enzyme inhibitors, which reduce proteinuria in NPS patients [5]. Therefore, we discontinued corticosteroids without the exacerbation of nephrotic syndrome.

## Conclusions

Patients with NPS may develop nephrotic syndrome prior to overt orthopedic symptoms and only show non-specific findings in renal biopsy at an early stage of NPS nephropathy. Additionally, hereditary nephrotic syndrome, often presenting as childhood-onset SRNS, may be difficult to diagnose in patients with the following conditions: renal symptoms prior to overt extrarenal symptoms, de novo mutations, and non-specific findings in renal biopsy. Therefore, in the management of SRNS in children, we need to reconsider the possibility of hereditary diseases such as NPS even without a family history.

## Abbreviations

FSGS: Focal segmental glomerulosclerosis
GBM: Glomerular basement membrane
LMX1B: LIM homeobox transcription factor 1 beta
NPS: Nail-patella syndrome
SRNS: Steroid-resistant nephrotic syndrome
